# Screening ciliopathy genes in the model organism *Trypanosoma brucei*

**DOI:** 10.1186/2046-2530-1-S1-P81

**Published:** 2012-11-16

**Authors:** S Barry, K Towers, S Vaughan

**Affiliations:** 1 Oxford Brookes University, UK

## 

We have carried out a bioinformatics study to search for novel proteins of the flagellum/cilium. A total of 26 candidate hypothetical genes were established from previously published studies including a flagellar proteome [1] and an RNAi study of motility mutants of *Trypanosoma brucei *[2]. All candidates are predicted to have orthologues in the human genome, the dysregulation of which is associated with or predicted to be involved in at least one ciliopathy. The protozoan parasite *T. brucei* is a well established experimental model to study defects in flagellum assembly and function. In this project we will confirm that the proteins from the candidate genes are localised to the flagellum or basal body by GFP-tagging. The function of each will be assessed using inducible RNAi methods and in future work will involve taking the work forward into human cell lines for some candidate genes to see if we can reproduce the same phenotype in the cilia of human cells.
